# High Transcript Levels of Vitamin D Receptor Are Correlated with Higher mRNA Expression of Human Beta Defensins and IL-10 in Mucosa of HIV-1-Exposed Seronegative Individuals

**DOI:** 10.1371/journal.pone.0082717

**Published:** 2013-12-05

**Authors:** Wbeimar Aguilar-Jiménez, Wildeman Zapata, Antonio Caruz, María T. Rugeles

**Affiliations:** 1 Grupo Inmunovirología, Facultad de Medicina, Universidad de Antioquia UdeA, Medellín, Colombia; 2 Grupo Infettare, Facultad de Medicina, Universidad Cooperativa de Colombia, Medellín, Colombia; 3 Unidad de Inmunogenética, Departamento de Biología Experimental, Facultad de Ciencias Experimentales, Universidad de Jaén, Jaén, España; Imperial College London, United Kingdom

## Abstract

Vitamin D (VitD) is an endogenous immunomodulator that could protect from HIV-1 infection reducing immune activation and inducing the expression of anti-HIV-1 peptides. To establish a correlation between VitD and natural resistance to HIV-1 infection, a case-control study using blood and mucosa samples of 58 HIV-1-exposed but seronegative (HESN) individuals, 43 HIV-1 seropositives (SPs) and 59 non-exposed healthy controls (HCs) was carried out. The VitD concentration in plasma was determined by ELISA, and mRNA relative units (RU) of VDR, IL-10, TGF-β, TNF-α and IL-1β in peripheral blood mononuclear cells (PBMCs), oral and genital mucosa was quantified by qRT-PCR. mRNA levels of human beta-defensin (HBD) -2 and -3 were previously reported and used for correlations. Significantly higher levels of VitD were found in plasma as well as higher mRNA RU of VDR in PBMCs, and in genital mucosa from HESN compared to HCs. In addition, higher mRNA RU of TNF-α, IL-1β and IL-10, and lower mRNA RU of TGF-β were found in PBMC from HESNs compared to HCs. We also observed higher IL-10 mRNA RU in genital mucosa of HESNs compared to HCs, and the mRNA levels of TNF-α in oral and genital mucosa of SPs were higher compared to HESNs. Furthermore, positive correlations between VDR and IL-10 mRNA RU in PBMCs and genital mucosa of HESNs were found. Finally, HBD-2 and HBD-3 mRNA RU were positively correlated with VDR mRNA expression in oral mucosa from HESNs. These results suggest that high levels of VitD and its receptor are associated with natural resistance to HIV-1 infection. Up-regulation of the anti-inflammatory IL-10, and the induction of anti-HIV-1 defensins in mucosa might be part of the mechanisms involved in this association. However, further studies are required to define causal associations.

## Introduction

Several mechanisms of protection against HIV-1 infection have been reported in HIV-1 highly exposed, but seronegative (HESN) individuals (Reviewed in 1); however, they do not explain the absence of infection in all HESNs. Therefore, further studies are required to fully understand the phenomenon of HIV-1 natural resistance to define new therapeutic targets. 

Beyond its role in mineral metabolism, vitamin D (VitD) has immunomodulatory effects [2,3]. Along with its receptor (VDR), they induce transcription of antimicrobial peptides [4] such as human beta defensins (HBD) that possess anti-HIV-1 activity *in vitro* [5], and play a potential protective role during exposure to HIV-1 in oral mucosa of the Colombian HESN population [6], (Zapata et al., 2012, submitted for publication). Moreover, VitD might reduce the immune activation and the number of viral target cells [2,3,7], further supporting its beneficial role in natural resistance to HIV-1 infection. 

In contrast, other studies have shown that the VitD/VDR complex enhances activation of the HIV-1 promoter located in the long terminal repeats [8,9]. Additionally, genetic variants related to high VitD and VDR function have been associated with susceptibility to HIV-1 infection [10], suggesting a potential pathogenic role of the VitD/VDR axis. 

These apparently contradictory data underline the importance of further studies on this topic, and suggest that the VitD pathway may have a dual role during HIV-1 infection. Particularly, VitD could be beneficial avoiding HIV-1 infection during virus exposure or reducing immune activation during the acute phase, the main pathogenic mechanism associated with this infection. In contrast, during chronic infection VitD could be detrimental, impairing the immune response against the virus. Therefore, to establish an association between VitD and natural resistance to HIV-1 infection, we compared the levels of i) plasma VitD; ii) VDR mRNA; iii) mRNA of the cytokines Interleukin (IL)-10, Transforming Growth Factor beta (TGB-β), Tumor Necrosis Factor alpha (TNF-α) and IL-1β, and iv) mRNA of the antimicrobial peptides HBD-2 and HBD-3 in peripheral blood mononuclear cells (PBMCs) and in mucosa samples of HESN, HC and SP individuals.

## Materials and Methods

### Population and samples

This is a cross-sectional study involving a cohort of Colombian sexual serodiscordant couples composed of 58 HESN and 43 chronically HIV-1-infected subjects, hereafter called seropositives (SPs); they were recruited from the HIV-1 comprehensive care programs in Santa Marta and Medellín, Colombia. We also included 59 non-exposed healthy control (HC) volunteers with similar demographic backgrounds as the HESN and SP individuals. The inclusion criteria for HESN subjects were previously reported [6,11]; briefly, the HESNs in this study had unprotected sexual intercourse with an SP partner more than five times monthly within at least 2 years of follow up, a negative HIV-1 ELISA test and no other infectious diseases at sampling. 

HC individuals have a negative HIV-1 ELISA test, fewer than 2 sexual partners in the last 2 years and self-reported no risk behaviors for HIV-1 infection. A complete demographic profile is shown in table 1.

**Table 1:** Demographic profile.

**Characteristics**	**HESN (n=58)**	**HC (n=59)**	**SP (n=43)**
**Age, mean years ± SD**	34.9 ± 10.3	32.8 ± 9.8	33.7 ± 7.1
**Males, n (%)**	24 (41.4)	25 (42.4)	23 (53.5)
**VL. median (interquartile range**)	…	…	2,569 (400 - 25,250)
**CD4+ T cell/µL count. median (interquartile range)**	ND	ND	357 (186 - 464)
**HAART-naïve, [n, %] [VL median (interquartile range)]**	…	…	[15, 35] [10,257 (718 - 23,188)]
**HAART-responders, [n, %] [VL maximum]**	…	…	[10, 23.3] [<400]
**HAART-non-responders [n, %] [VL median (interquartile range)]**	…	…	[18, 41.9] [35,806 (18,200 – 118,770)]

HESN: HIV-1 exposed seronegative; HC: Healthy controls; SP: Seropositives, SD: Standard deviation; VL: Viral Load (copies/mL); ND: Not determined.

Furthermore, to rule out stratification bias we defined the principal component of ancestry of SPs and HESNs by determining and comparing the frequency of 17 single nucleotide polymorphisms used as ancestry informative markers (delta value ≥ 0.40 [12]) with the reported frequencies in samples from Africa and Europe (1000 genome catalog: http://browser.1000genomes.org/index.html).

All individuals signed an informed consent prepared according the Colombian Legislation and this study was approved by the Bioethics Committee, Universidad de Antioquia. 

Mucosal samples from oral, vaginal and endocervical mucosa were taken from all individuals using a cytobrush, as previously described [6]. Blood from 23 HESNs, 38 HCs and 23 SPs was obtained and plasma was separated by blood centrifugation at 380 x g for 10 min; peripheral blood mononuclear cells (PBMCs) were isolated by gradient centrifugation at 400 x g for 30 min using Ficoll-Hypaque (Sigma), following the manufacturer’s instructions. In addition, mRNA relative units (RU) of HBD-2 and HBD-3 sampled from the mucosa of this serodiscordant cohort in a previous study (Zapata et al., 2012, submitted for publication), were used to correlate them with VDR mRNA expression.

### Plasma vitamin D and VDR mRNA quantification

Plasma VitD [25(OH)D] levels were quantified by an electrochemiluminescence immunoassay (Cobas - Roche), following the manufacturer’s instructions.

Total RNA was extracted from PBMCs and mucosal samples using TRizol Reagent (Invitrogen, Carlsbad, CA) following the manufacturer’s instructions. RNA was treated with DNase I and Ribolock (Thermo Scientific, St. Leon-Rot, Germany) and stored at -70°C until used as previously reported [6].

cDNA was synthesized using random hexamers and the Revertaid H Minus Retrotranscriptase (Thermo Scientific) following the manufacturer’s instructions. 

Quantitative real time PCR (qPCR) was performed using 15 µL final volume of 2 µL cDNA, 1X Maxima probe qPCR master mix kit (Thermo Scientific) and 0.35X of hydrolysis probes (TaqMan®, from Applied Biosystems), for VDR (Hs01045844_m1), and the reference genes β-actin (4310881E) and phosphoglycerate kinase 1 (PGK1) (Hs00943178_g1).

In addition, 1X Maxima SYBR green qPCR master mix kit (Thermo Scientific) and 260 µM of specific primers for IL-10 (Fw: 5’-GCTGAGAACCAAGACCCAGAC-3’ and Rv: 5’-GGAAGAAATCGATGACAGCG-3’), TGF-β (Fw: 5’-CAGCAACAATTCCTGGCGATA-3’ and Rv: 5’-AAGGCGAAAGCCCTCAATTT-3’), TNF-α (Fw: 5'-CCCATGTTGTAGCAAACCCTC-3' and Rv: 5'-TATCTCTCAGCTCCACGCCA-3'), IL-1β (Fw: 5'-CTCGCCAGTGAAATGATGGCT-3' and Rv: 5'-GTCGGAGATTCGTAGCTGGAT-3') and the reference genes β-actin (Fw: 5'-CTTTGCCGATCCGCCGC-3' and Rv: 5'-ATCACGCCCTGGTGCCTGG-3') and PGK1 (Fw: 5'-GTTGACCGAATCACCGACC-3' and Rv: 5'-TCGACTCTCATAACGACCCGC-3') were used. The cycling profiles in all experiments were: 95°C for 10 min, followed by 40 cycles at 94°C for 15 sec, and annealing/extension for 1 min at 60°C. Relative expression units of mRNA (RU) was calculated by the ΔCt method [13], using the expression of β-actin and PGK1 to normalize the amount of RNA in the samples. The Bio-Rad CFX manager 3.0 (Bio-Rad) was used to acquire the cycles thresholds that were determined in each sample using a regression fit in the linear phase of the PCR amplification curve. Triplicate assays were performed (SD were less than 0.3 cycles in all assays). The results are given as median of relative expression units to two reference genes. 

### Statistical analysis

The categorical variables in different groups of individuals were compared using the chi-square test. According to bivariate normality assumption through the Shapiro–Wilk test, a two-tailed parametric t-test was used to compare the levels of 25(OH)D, and a non-parametric test (Mann-Whitney *U*-two-tailed test) to compare the relative units of the mRNAs of VDR, IL-10, TGF-β, TNF-α and IL-1β between HESN and both, SP and HC individuals. A two-tailed *p* value <0.05 was considered statistically significant; furthermore, we used a Bonferroni test for adjusting by the number of comparisons. The correlations between VDR mRNA and viral load, 25(OH)D, transcript levels of IL-10, TGF-β, TNF-α, IL-1β, HBD-2 or HBD-3 were evaluated using the Spearman coefficient rank (r). The statistical tests were performed using the GraphPad Prism version 6.0 

## Results

### Demographic data

We analyzed samples of 58 HESN, 43 SP and 59 HC, and no significant differences were found regarding demographic backgrounds among the groups (Table 1). The median viral load of SP individuals was 2,569 RNA copies/mL (interquartile range= 400–25,250 copies/mL) and the HESNs showed a monthly average of 8 unprotected sexual oral/anal/vaginal intercourse events with their SP partner within 2 years of enrollment, suggesting a moderate risk of acquiring the virus. In addition, 47% of SP and 27% of HESN reported a previous sexually transmitted disease. Finally, although the majority (80%) of sexual orientation in the serodiscordant cohort was heterosexual, bisexual orientations was also present (20%). In addition, a similar ancestry component, and pair-wise fixation index (FST) values indicated that our discordant couples were not stratified as previously shown [14].

### Higher vitamin D levels and VDR mRNA expression are associated with resistance to HIV-1 infection

To determine whether plasma 25(OH)D levels and transcripts of VDR could be associated with natural resistance to HIV-1 infection, their expression was compared between HESN and both, SP and HC individuals.

**Figure 1:**
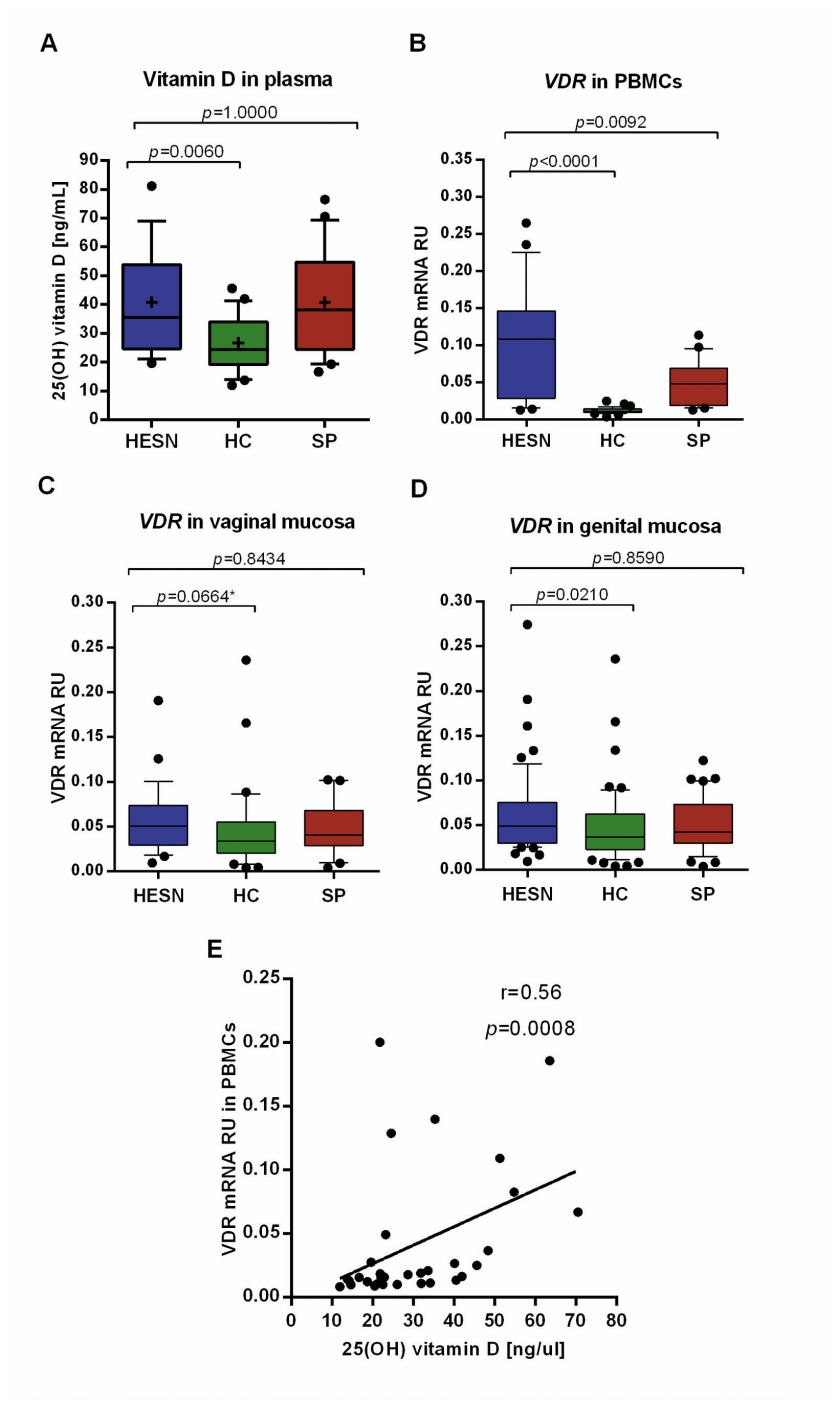
High Vitamin D levels and *VDR* mRNA expression in HESN individuals. The box and whisker plots show significantly higher levels of 25(OH)D levels in plasma (A), relative mRNA units, RU, of VDR in PBMCs (B), vaginal (C) and genital mucosa (vaginal and endocervical mucosa, together) (D) of HESNs (blue) compared to HCs (green). Significantly higher levels of VDR RU in PBMCs of HESNs than in SPs (red) are also shown (B). Plasma levels of 25(OH)D from all populations was positively correlated with VDR mRNA RU in PBMCs (r=0.56, p=0.0008, n=32) (E). VDR mRNA RU was obtained by normalizing it with β-actin and phosphoglycerate kinase 1 (PGK1) mRNA expression levels. Box: 25–75 percentile range; whiskers: 10–90 percentile range; outliers are plotted as black circles; medians are the black lines in the boxes and means plotted as a plus and Bonferroni adjusted significant *p* values are displayed in each graph. A two-tailed parametric t test was used to compare the levels of 25(OH)D, and the non-parametric test (Mann-Whitney *U*-two-tailed test) to compare VDR mRNA RU between pairs of groups. The correlations were evaluated using the Spearman coefficient rank (r). *unadjusted *p*-value=0.0332.

Significantly higher 25(OH)D levels were found in plasma of HESNs compared to HCs (mean±SD; 40.81±18.40 vs. 26.69±9.64 ng/mL, respectively, *p*=0.0060; Figure 1a); no significant differences in 25(OH)D levels were observed between HESNs and SPs (*p*=1.0000; Figure 1a).

The VDR mRNA expression in PBMCs, oral, vaginal and endocervical mucosa was then compared. Significantly higher VDR mRNA expression occurred in HESNs vs. HCs in PBMCs (median [interquartile range] 0.108 [0.028 - 0.146] vs. 0.011 [0.009 - 0.014], respectively, *p*<0.0001, Figure 1b), and in genital mucosa analyzed together as vaginal and endocervical mucosa (0.049 [0.030 - 0.075] vs. 0.036 [0.023 - 0.062], respectively, *p*=0.0210. Figure 1d). Likewise, higher VDR mRNA RU were observed in vaginal mucosa of HESNs compared to HCs (0.051 [0.029 - 0.074] vs. 0.034 [0.020 - 0.055], respectively, *unadjusted p*=0.0332. Figure 1c), but the statistical significance was lost after the Bonferroni correction (*p*=0.0664, Figure 1c). In addition, HESNs showed significantly higher VDR mRNA levels compared to SPs in PBMCs (0.108 [0.028 - 0.146] vs. 0.048 [0.019 - 0.069], respectively, *p*=0.0092. Figure 1b), but no significant differences between HESNs and SPs were found in mucosa samples (Figure 1c, 1d). Finally, no significant differences in VDR mRNA expression levels in oral or endocervical mucosa were detected between HESN and HC or SP individuals (data not shown). We also analyzed the data stratified by gender, and no significant differences were found.

In addition, a positive correlation between plasma levels of 25(OH)D and levels of VDR mRNA in PBMCs was detected (r=0.56, *p*=0.0008, Figure 1e).

Finally, since VDR levels as observed in PBMCs and genital mucosa of HESN and SP individuals could be induced by viral exposure, correlations between viral load and VDR mRNA levels in SP individuals was evaluated; however, no significant correlation was found (r=0.33, *p*=0.2274).

### HESNs exhibit an up-regulation of IL-10 than HCs in mucosa, maintaining similar levels of TNF-α, IL-1β and TGF-β

As VitD displays anti-inflammatory properties [2,3,7] with a potential impact reducing immune activation, the main pathogenic mechanism in HIV-1 infection, the transcriptional expression of pro-inflammatory (IL-1β and TNF-α) and anti-inflammatory (IL-10 and TGF-β) cytokines in PBMCs and mucosa were evaluated to infer the degree of immune activation. 

**Figure 2:**
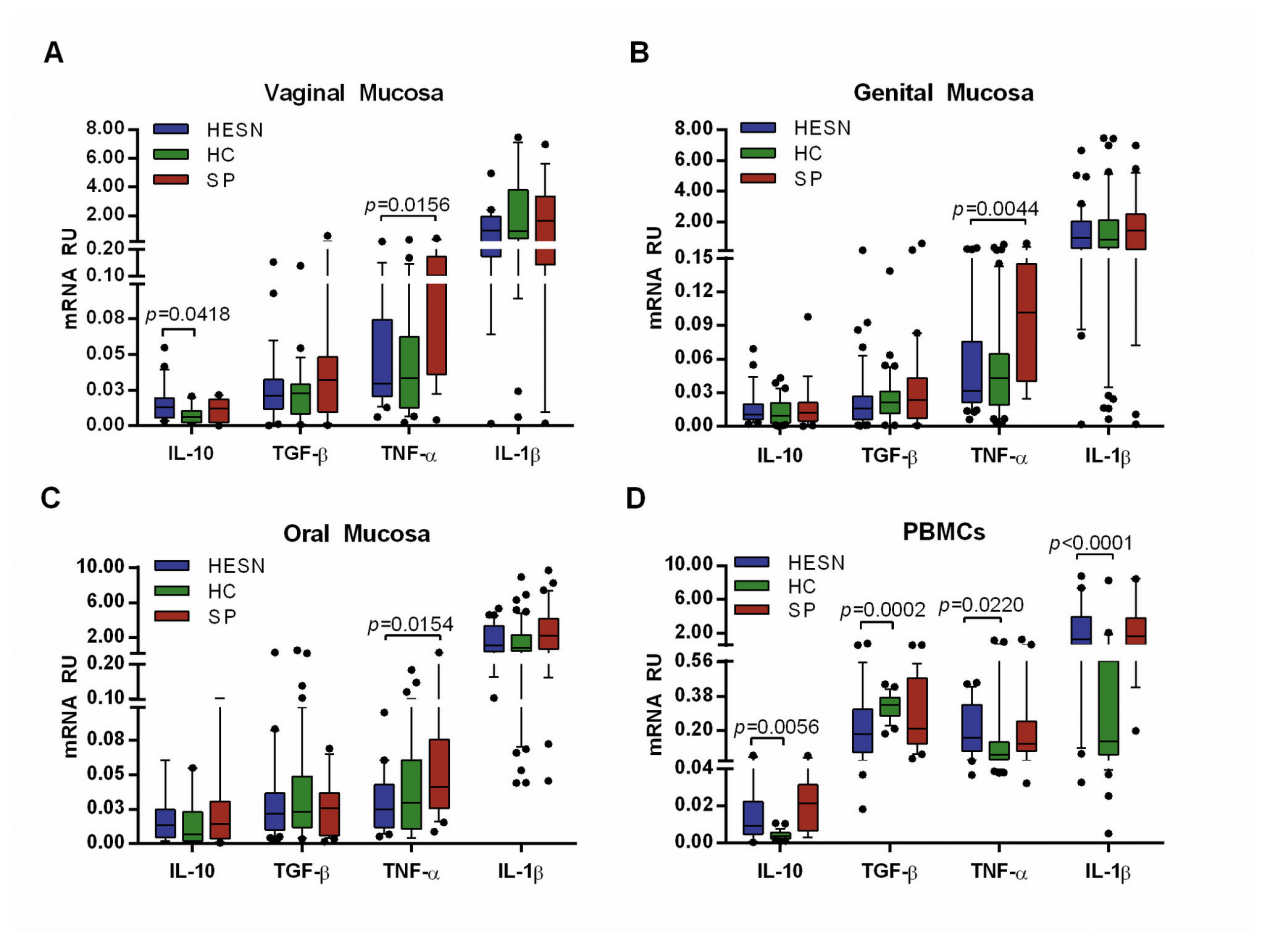
Activation status of HESNs is variable comparing mucosa, versus PBMCs. The box and whisker plots show the median value and 10-90 percentiles of mRNA RU of anti-inflammatory IL-10, TGB-β and pro-inflammatory cytokines TNF-α and IL-1β in vaginal mucosa (A), genital mucosa (vaginal and endocervical mucosa, together) (B), oral mucosa (C) and in PBMCs (D) of HESNs (blue), HCs (green) and SPs (red). A non-parametric test (Mann-Whitney *U*-two-tailed test) was used to compare mRNA RU between study groups. Outliers are plotted as black circles and Bonferroni adjusted significant *p* values are displayed in each graph. Significantly lower mRNA RU of TNF-α in vaginal, genital and oral mucosa of HESNs than SPs (A-C) and significantly higher mRNA RU of IL-10 in vaginal mucosa (A) and PBMCs (D) of HESNs than HCs are shown. In contrast, the PBMCs of HESNs showed higher levels of the proinflammatory cytokines TNF-α and IL-1β (D).

Higher levels of IL-10 transcripts in vaginal mucosa of HESNs compared to HCs (0.013 [0.006 - 0.019] vs. 0.006 [0.002 - 0.011], respectively, *p*=0.0418. Figure 2a) were observed, whereas, higher levels of TNF-α were found in SPs compared to HESNs in vaginal (0.102 [0.036 - 0.173] vs. 0.030 [0.021 - 0.074], respectively, *p*=0.0156. Figure 2a), genital (vaginal and endocervical mucosa, together) (0.102 [0.040 - 0.145] vs. 0.031 [0.021 - 0.075], respectively, *p*=0.0044. Figure 2b) and oral mucosa (0.041 [0.026 - 0.076] vs. 0.025 [0.012 - 0.043], respectively, *p*=0.0154. Figure 2c). Furthermore, the expression levels of TNF-α or IL-1β in mucosa between HESNs and HCs were similar (Figure 2a,b,c).

In contrast, the mRNAs of pro-inflammatory cytokines TNF-α and IL-1β were higher in PBMCs of HESNs compared to HCs (TNF-α: 0.161 [0.092 - 0.335] vs. 0.072 [0.049 - 0.140], respectively, *p*=0.0220 and IL-1β: 1.287 [0.569 - 3.947] vs. 0.145 [0.073 - 0.565], respectively, *p*<0.0001. Figure 2d). No significant differences were observed between HESNs and SPs (Figure 2d). In addition, TGF-β mRNA levels in PBMCs were lower in HESNs than in HCs (0.182 [0.087 - 0.312] vs. 0.335 [0.279 - 0.374], respectively, *p*=0.0002. Figure 2d) but no significant differences in TGF-β mRNA in PBMCs or mucosa were detected between HESNs and SPs (Figure 2). Furthermore, IL-10 mRNA levels were higher in PBMCs of HESNs compare to HCs (0.010 [0.005 - 0.023] vs. 0.004 [0.003 - 0.005], respectively, *p*=0.0056. Figure 2d). 

**Figure 3:**
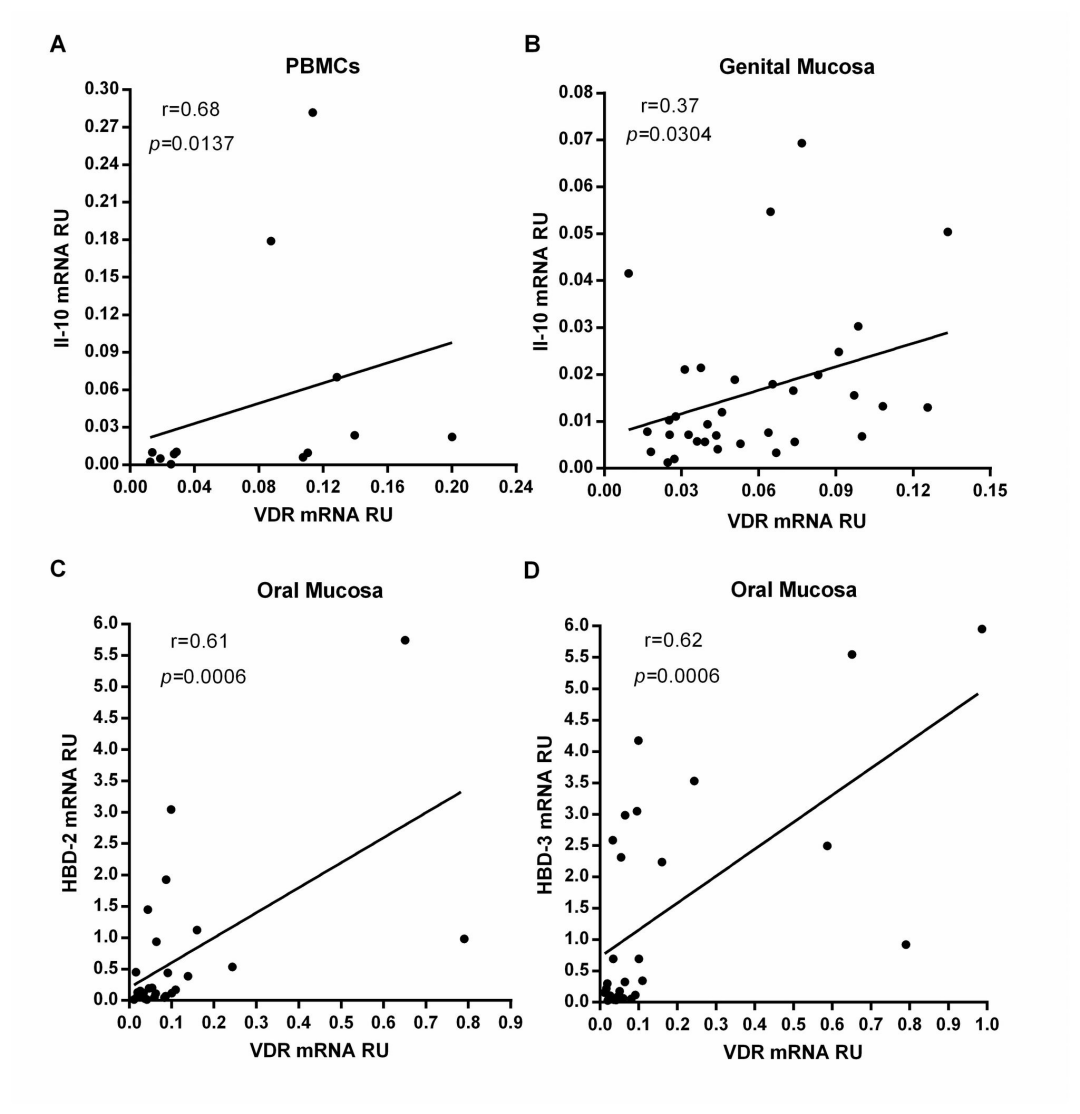
VDR mRNA expression is correlated with mRNA of IL-10 and antimicrobial peptides (HBD-2 and HBD-3) in HESNs. There was a significant positive correlation between the mRNAs of VDR and the IL-10 in PBMCs (r=0.68; *p*=0.0137, n=13) (A), and in the genital mucosa (r=0.37; *p*=0.0304, n=34) (B) of HESN individuals. There were also significant positive correlations between the mRNAs of VDR and the antimicrobial peptides HBD-2 (r=0.61, *p*=0.0006, n=28) (C), and HBD-3 (r=0.62, *p*=0.0006, n=27) (D) in oral mucosa of HESN individuals. The correlations were evaluated using the Spearman coefficient ranks (r), which are displayed in each graph with the best linear fit lines and *p* values.

Despite all cytokines mRNA levels were correlated with VitD and VDR mRNA within each cohort, the only significant correlation found in the HESN cohort was between VDR mRNA and IL-10 mRNA in PBMCs (r=0.68; *p*=0.014. Figure 3a) as well as in genital mucosa (r=0.37; *p*=0.0304. Figure 3b). 

Finally, no major differences in the evaluated cytokines in neither blood nor mucosa compartments were observed among HAART naïve and patients on HAART (responders and non-responders), suggesting that all SP individuals have similar level of immune activation regardless viral load. 

### VDR mRNA expression was correlated with mRNA of antimicrobial peptides (HBD-2 and HBD-3) in oral mucosa of HESNs

Previously, we showed that the mRNA of the antimicrobial peptides HBD-2 and HBD-3 are expressed in greater amounts in oral mucosa of the same HESN individuals [6], (Zapata et al., 2012, submitted for publication). Therefore, correlations between the mRNA levels of VDR and the antimicrobial peptides HBD-2 and HBD-3 were evaluated. Significant positive correlations in oral mucosa of HESNs (r=0.61, *p*=0.0006 for HBD-2 and VDR; r=0.62, *p*=0.0006 for HBD-3 and VDR; Figure 2c,d) and in oral samples of all individuals (HESN, SP and HC) (r=0.46, *p*<0.0001 for HBD-2 and VDR; r=0.44, *p*<0.0001 for HBD-3 and VDR) were observed. No significant correlations in genital mucosa were found (data not shown).

## Discussion

Defining the immune factors involved in natural protection against HIV-1 infection in HESN individuals is a key goal for the development of vaccines or new therapeutic tools. 

The VitD/VDR axis has strong immunomodulatory and antimicrobial effects [2,4] and could play an important role modulating the risk of HIV-1 infection; however, reports on the influence of this axis on HIV-1 infection are contradictory.

We analyzed a cohort of Colombian HESNs, one of the best characterized HESN cohort so far studied in South America, and significantly higher plasma 25(OH)D levels were detected compared to HCs (Figure 1a). 

Most importantly, we found significantly higher VDR mRNA expression levels in PBMCs as well as in genital mucosa, the primary HIV-1 port of entry [15,16], of HESNs compared to HCs (Figure 1b,d). However, when the analysis was carried out in endocervical and vaginal mucosa independently, the statistical differences were not significant for endocervical mucosa (*unadjusted p*=0.1589 Figure 1c) or the significance was lost after the Bonferrini correction for vaginal mucosa (*unadjusted p*=0.0332 and adjusted *p*=0.0664. Figure 1c); these results are most likely explained by the reduction in the sample size. The fact that significant differences in mucosa were less robust than those in PBMC samples might be due to compartmentalization issues, and differences in cell subpopulations: immune cells in peripheral blood versus epithelial cells in mucosa.

Furthermore, a positive correlation between plasma levels of 25(OH)D and levels of VDR mRNA in PBMCs was detected (Figure 1e), suggesting that high levels of VitD could induce the expression of VDR as previously reported in *in vitro* experiments [2,17]. 

To the best of our knowledge, this is the first study reporting an association between VitD and VDR levels in circulating immune cells as well as in mucosa samples with natural resistance to HIV-1 infection. While early studies presented contradictory results related to the *in vitro* effect of VitD on HIV-1 infection [18,19], a recent study reported that VitD could inhibit HIV-1 infection through autophagy induction [20], highlighting the beneficial role of VitD by a new proposed mechanism. Whether autophagy is one of the VitD-induced protective pathways in HESNs is unknown; thus additional studies exploring this specific point are required. In addition, diet, skin color and exposure to sun are aspects influencing 25(OH)D levels [21,22]. Therefore, these should be considered in further studies.

The possibility that the expression of VDR is virus-induced is unlikely since no significant correlation was found between VDR mRNA levels and viral load (r=0.33, *p*=0.2274), therefore, our results may indicate a genetic predisposition of HESNs to up-regulate VDR expression upon an immune challenge. Although, allelic variants in the *VDR* gene have been previously reported in HESN individuals [10], the direct influence of these variants on 25(OH)D or VDR expression in HESNs has not been explored. Therefore, to clarify these findings, it is essential to genotype variants in genes of the VitD pathway and correlate them with 25(OH)D and VDR expression; such studies are currently undertaken in our laboratory. 

During infections, the production of cytokines and chemokines is increased, allowing migration and activation of effector cells. However, to date it is not clear if this phenomenon can be detrimental by increasing target cells [23–26] or protective against HIV-1 infection by promoting vigorous and effective immune responses that could limit viral infection [27–29]. In this study, higher levels of IL-10 transcripts in vaginal mucosa of HESNs compared to HCs were observed (Figure 2a); moreover, while SPs demonstrated a substantially increased production of TNF-α in both the genital tract and oral mucosa compared to HESN individuals, the expression levels of TNF-α and IL-1β in mucosa of HESNs and HCs were similar (Figure 2a,b,c). These results, suggest that despite viral exposure, the HESNs, may control the production of pro-inflammatory cytokines, which is in accordance with previous studies indicating lower levels of activation in mucosa tissues of HESNs [23–26]. In contrast, the HESNs expressed a mixed pattern of pro- and anti-inflammatory cytokines in PBMCs: they exhibited significantly higher amounts of mRNA of TNF-α, IL-1β and IL-10 and lower amounts of mRNA of TGF-β compared to HCs (Figure 2d). The difference in mucosa and blood compartments may be explained by the fact that the mucosa usually exhibits a high regulatory environment required to prevent tissue damage as a result of constant immune challenges, whereas PBMCs include circulating cell subsets with various phenotypes and different activation levels raised from tissues with diverse antigenic microenvironment. Considering that individuals of the HESN cohort are sexually exposed to HIV-1, the mucosa became the most important tissue in this study, where critical events such as proinflammatory milieu, the number of viral target cells or preexisting protective factors may determine the result of HIV-1 exposure.

Furthermore, a significant correlation between VDR mRNA and IL-10 mRNA were detected in PBMCs (Figure 3a) and in genital mucosa (Figure 3b) of HESNs. The relationship between high VDR expression and high IL-10 production in HESNs is not surprising since a VitD response element (VDRE) in the *IL-10* gene promoter has been identified, that elicits an increase in IL-10 expression after treatment with VitD [30]. Likewise, it has been demonstrated that VitD induces IL-10 expression in monocytes and CD4+ T cells *in vitro* [2,31], targeting epidermal and dermal dendritic cells for the induction of distinct regulatory T cells, including T regulatory cells 1 (TR1) and IL-10 secreting cells [32], and reducing the inflammation by decreasing pro-inflammatory cytokines such as IL-1β and TNF-α [33,34]. Moreover, increased IL-10 secreting cells and IL-10 expression is also seen in humans supplemented with VitD [35,36], suggesting that VitD/VDR complex in HESNs might up regulate the expression of IL-10, and eventually reduce immune activation, at least locally.

Since the VitD/VDR complex promotes the expression of antiviral proteins [4,37–39], we next investigated whether high expression of VDR is correlated with the expression of the antimicrobial peptides, HBD-2 and HBD-3, in the mucosa of HESNs; indeed, a significant positive correlation between mRNA levels of VDR and HBD-2 and HBD-3 in oral mucosa of HESNs (Figure 2c,d) was observed; furthermore, significant positive correlations in oral samples of all individuals were also found.

Previous reports have shown that treatment with VitD of several cell types including epithelial cells, induced along with VDR, a dose-dependent production of catelicidin, HBD-2 and HBD-3 antimicrobial peptides [4,17,38,40,41]. This response restricted the growth of pathogens such as *Aggregatibacter actinomycetemcomitans* [17] and *Staphylococcus aureus* [41] in the mucosa and oral cavity, enhancing the innate immune defenses without increasing inflammation [17,41]. Moreover, oral transmission is a potentially important, yet less well understood route of HIV-1 infection, where HBDs seem to be involved in the low rate of oral HIV-1 transmission [42,43]. Since no correlation was detected between VDR and HBDs mRNA in genital samples, the potential protective role of VDR through the induction of these antiviral peptides is so far limited to oral mucosa, pointing to the requirement of further studies to confirm if VDR-induced HBD expression protects from HIV-1 infection in genital mucosa. 

Overall these results suggest that VitD and VDR could influence the resistant phenotype exhibited by this highly HIV-1 sexually exposed but seronegative Colombian cohort; the most likely VitD-induced mechanisms involved in avoiding the establishment of HIV-1 infection would be the up-regulation of the anti-inflammatory IL-10, and/or the induction of the antimicrobial peptides HBD-2 and HBD-3 with anti-HIV-1 activity. Considering that the cytokine profile in HESNs’ PBMCs is different from the one in mucosa, a compartmentalization effect could be taking place. Since the cross-sectional design of our study limits causal conclusions, these findings need to be confirmed by *in vitro* assays. 
